# 

**Published:** 1902-09-20

**Authors:** 


### NOTICE TO CORRESPONDENTS.

All MSS., Letters, Books for Review, and other matters intended for the Editor should be addressed The Editor, "The Hospital," The Hospital Building, 28 & 29 Southampton Street, Strand, W.C.

The Editor cannot undertake to return rejected MS., even when accompanied by stamped directed envelope.

**Notice to Advertisers and Subscribers.**

All Advertisements, Orders for Copies of The Hospital, and Business Communications should be addressed to **THE MANAGER, (Not to the Editor)**, The Hospital, 28 & 29 Southampton Street, Strand, London, W.C.

The Cost of Subscription, post free, is as follows.—Per week, 2½d.; per quarter, 2s. 8d.; per half-year, 5s. 3d.; per annum, 10s. 6d. Foreign:—Per annum, 15s. 2d., payable, in all cases, in advance.

**NOTICE.—Vol. XXXI. of "The Hospital" (October 1901, to March 1902), in handsome cloth binding is now on sale at the office of this Journal, price, 7s. 6d. Cases for binding the Numbers contained in this Volume 1s. 6d. Index to Vol. XXXI., 2½d., post free.**

**The Monthly Part for September is now ready. Price 6d., by post 8d.**

### BOOKS RECEIVED.

R. Brimley Johnson.

"Proceedings of the Society for Psychical Research."

The Walter Scott Publishing Company.

"The Wife and Mother," a book of first principles for the guidance of young married women. By Ralph Vincent, M.D.

The Bible House.

"The Book of God's Kingdom."

P. S. King and Son.

"The London Life Table, 1891–1900." By Shirley F. Murphy.

"The Thirteenth Annual Report of the Asylums Committee of the London County Council, and of various Sub-committees."

### The Student of Medicine.

### APPOINTMENTS.

**W. E. Barker**, M.B., Ch.B.Vict., appointed Medical Officer of Health for Clitheroe Urban District. **H. A. Clark**, M.B., C.M Edin., appointed Medical Officer and Public Vaccinator for the Drax and Carlton Districts of the Selby Union. **John Crow**, M.B., Ch.B.Glas., appointed Certifying Factory Surgeon for the Largs District of Ayrshire. **S. F. Crowther-Smith**, M.B.Lond., M.R.C.S., appointed District Medical Officer of the Alton Union. **Henry E. N. Dobie**, M.B., appointed Public Vaccinator for the Midland District, Victoria. **Hubert de Burgh Dwyer**, M.R.C.S., L.R.C.P.Lond., appointed Medical Officer to the Banbury Union Workhouse Infirmary. **H. Byam Ellerton**, M.R.C.S., L.R.C.P., appointed Senior Assistant Medical Officer to the Metropolitan Asylum, Leavesden, Watford, Herts. **Norman Edward Gibbs**, L.R.C.P., L.R.C.S Edin., L.F.P.S.Glasg., appointed House Surgeon to the West Kent General Hospital, Maidstone. **John Gordon**, M.D., B.S.Melb., F.R.C.S.Eng., appointed Honorary Surgeon to Out-patients of the Melbourne Hospital, Victoria. **John Henderson**, M.D.Glasg., appointed Extra Dispensary Physician to the Glasgow Royal Infirmary. **Charles H. Johnson**, M.D., appointed Health Officer for the Shire of Yacandondah. **J. D. Kenny**, M.D, M.Ch.R.U.I., appointed District Medical Officer to the Rotherham Union. **W. Spalding Laurie**, M.B., B.S.Melb., appointed House Physician to the Perth Public Hospital, Western Australia. **W. K. Parbury**, M.R.C.S., L.R.C.P.Lond., appointed District Medical Officer of the Bedford Union. **James A. N. Scott**, M.D.Glasg., F.R.C.S., appointed Health Officer for the Shire of Wycheproof, Victoria. **Lewis A. Smith**, M.D.Lond., M.R.C.P., appointed Assistant Physician to the London Hospital. **R. R. Stowell**, M.B.Melb., appointed Indoor Physician to the Children's Hospital, Melbourne, Victoria. **W. W. Stevens**, M B., Ch.M.Syd., appointed Junior Medical Officer to the Coast Hospital, New South Wales. **E. Treharne**, L.R.C.P., L.R.C.S.Edin., L.F.P.S.Glasg., appointed District Medical Officer of the Cardiff Union. **Ernest C. D. Walker**, M.B., C.M., appointed Assistant Medical Officer of the Seacliff Lunatic Asylum, New Zealand. **Charles Henry Wheeler**, M.D., appointed a Port Health Officer for the Port of Hokianga, New Zealand.

### "The Hospital" Institutional Library.

"Hospitals and Asylums of the World." 4 Vols., with a Portfolio of Plans, complete £12 12s.

"Burdett's Hospitals and Charities, 1902." 5s.

"Cottage Hospitals, General, Fever, and Convalescent." 10s. 6d.

"Hospital Expenditure: The Commissariat." 2s. 6d.

"A Legal Handbook for the Use of Hospital Authorities." 2s. 6d.

"The Cottage Hospital Case Book or Register of Patients." 10s. and 12s. 6d.

"The Furnishing and Appliances of a Cottage Hospital." 6d.

All these are published by the Scientific Press, Ltd., and may be obtained through any bookseller, or direct from the publishers, 28 and 29 Southampton Street, Strand, London, W.C.

